# A new directionality index based on high-resolution joint symbolic dynamics to assess information transfer in multivariate networks

**DOI:** 10.3389/fnins.2025.1504161

**Published:** 2025-02-13

**Authors:** Steffen Schulz, Andy Schumann, Karl-Jürgen Bär, Jens Haueisen, Georg Seifert, Andreas Voss

**Affiliations:** ^1^Charité Competence Center for Traditional and Integrative Medicine (CCCTIM), Charité – Universitätsmedizin Berlin, Corporate Member of Freie Universität Berlin, Humboldt-Universität zu Berlin and Berlin Institute of Health, Berlin, Germany; ^2^Lab for Autonomic Neuroscience, Imaging and Cognition (LANIC), Department of Psychosomatic Medicine and Psychotherapy, Jena University Hospital, Jena, Germany; ^3^Institute of Biomedical Engineering and Informatics, University of Technology Ilmenau, Ilmenau, Germany

**Keywords:** directionality, high-resolution joint symbolic dynamics, coupling analysis, causality, network physiology

## Abstract

The detection and quantification of coupling strength and direction are important aspects for achieving a deeper understanding of physiological regulatory processes in the field of network physiology. Due to the limitations of established approaches, we developed directionality indices based on simple mathematical symbolization principles and simple computational procedures that allow a quick and comprehensive understanding of the underlying couplings. We introduced a new directionality index (*D*_HRJSD_) derived from the pattern family density matrix of the High-Resolution Joint Symbolic Dynamics (HRJSD) approach and its multivariate version (mHRJSD) to determine coupling direction and driver-response relationships. The mHRJSD approach contains the multivariate directionality index *D*_mHRJSD_ (*D*_mHRJSD_(*x*,*y*|*z*), *D*_mHRJSD_(*x*,*z*|*y*), and *D*_mHRJSD_(*y*,*z*|*x*)), allowing us to determine the primary driver **D_mHRJSD_, the secondary driver *D_mHRJSD,_ and the dominant responder ^−^*D*_mHRJSD_ in multivariate systems that are at least weakly coupled. Different linear and non-linear bi- and multivariate coupled systems (Gaussian autoregressive models) with different mutual influences were generated to validate these indices. The simulation results showed that *D*_HRJSD_ was able to correctly detect the dominant coupling direction in linear bivariate coupled systems but was partly able to detect the dominant coupling direction in non-linear bivariate coupled systems. The proposed directionality index *D*_mHRJSD_ derived from the mHRJSD approach is able to correctly detect the driver-responder relationships in linear coupled systems. The main advantages of the newly introduced directionality indices include their insensitivity to non-stationary time series, their ability to capture couplings through a simple, fast, and easy-to-implement symbolization procedure, and their scale invariance. Additionally, they are independent of time series length, model order selection, and the procedure for determining their significance level.

## 1 Introduction

The complex interplay between the central nervous system (CNS) and autonomic nervous system (ANS) with their large number of subsystems (parasympathetic and sympathetic activity) is also known as the central autonomic network (CAN) (Bashan et al., 2012; Bartsch et al., 2015; Ivanov et al., 2016). It has been shown that the output of CAN is directly linked to ANS (heart rate) as well and that sensory information from peripheral end organs provides feedback to the CAN (i.e., baroreceptor reflex). Information transfer between the CNS and ANS operates as a feedback-feedforward system, dynamically responding to the body’s significant demands. These brain-heart interactions are involved in multiple bodily processes, including sensing, integration, and activity regulation, to maintain homeostasis (Craig, 2002). Communication between the brain and the heart is bidirectional and occurs through different neural mechanisms, such as the vagal and spinal pathways (Chen et al., 2021). Moreover, the relationships between cortical network segregations and cardiac dynamics as cardiac sympathetic–vagal oscillations may provide valuable insights into the affective state of healthy and diseased-related alterations in network physiology (Candia-Rivera et al., 2024).

The role of the cerebral cortex in autonomic control of the cardiovascular system is gaining increased attention in medicine. Different cardiovascular control centers in the brainstem deal with different reflex mechanisms of cardiovascular adjustment (i.e., the cardiopulmonary reflex, the chemoreflex, and the baroreflex) (Dampney, 1994). Here, neurons in the caudal and rostral ventrolateral medulla affect efferent sympathetic reflexes and contribute to the maintenance of heart rate and blood pressure via the intermediolateral cell column of the spinal cord. The two medullary areas, the ambiguous nucleus and the vague nerve’s dorsal motor nucleus, are preganglionic parasympathetic neurons mediating the efferent parasympathetic reflex mechanism (McAllen, 1976; Taylor et al., 2001).

The parasympathetic nervous system is responsible for the “rest and digest” function while sitting, resting, and relaxing. It constricts the pupils, slows the heart rate and contractility, contracts the bronchial musculature, stimulates bronchial secretions, and enhances gut motility for digestion. The preganglionic neurons synapse with postganglionic neurons in the parasympathetic ganglion located next to or in the effector end organs. The sympathetic nervous system dominates during “fight-or-flight” reactions and during exercise and thus prepares the body for stressful physical activity. Sympathetic nervous activity increases the flow of well-oxygenated blood and is rich in nutrients to the tissues that need it, particularly the working skeletal muscles. The preganglionic sympathetic neurons arise from the thoracic and lumbar regions of the spinal cord (segments T1 through L2) and are located about halfway between the CNS and the effector tissue (McCorry, 2007). The preganglionic neurons of both the sympathetic and parasympathetic divisions release the neurotransmitter acetylcholine. The postganglionic neurons of the parasympathetic system also release acetylcholine, whereas the postganglionic sympathetic neurons release norepinephrine (Rea, 2016). The cardiac or respiration-related activity (parasympathetic) is connected to preganglionic neurons. It has been shown that brain regions like the insula, thalamus, hypothalamus, amygdala, and medial prefrontal cortex are involved in autonomic regulation at rest and during cognitive or emotional stress conditions, proven by functional brain imaging (Ziegler et al., 2009; Shoemaker et al., 2015). Beissner et al. (2013) showed that largely divergent brain networks were associated with sympathetic and parasympathetic activity. The ventromedial prefrontal cortex (VMPFC), the perigenual anterior cingulate cortex (pACC), the dorsal anterior cingulate cortex (dACC), the posterior cingulate cortex (PCC), the insular cortices, and the amygdala seem to be the main cortical and subcortical areas involved in ANS regulation processes that are created by a network of interactions related to the task and autonomic division.

Investigating the coupling between these ANS subsystems, their variability, and brain activity may lead to a better understanding of pathophysiological regulatory processes within the central autonomic network. For the quantitative analysis of the brain-heart (CNS-ANS) network coupling pathways and its integrated interacting subsystems, such as the cardiovascular and cardiorespiratory systems, several linear/non-linear univariate and multivariate approaches are available.

These approaches focus on characterizing the multivariate information transfer. These concepts (Schulz et al., 2013a; Bartsch et al., 2015; Faes et al., 2015; Ivanov et al., 2016) are applicable in the following domains: entropy, Granger causality, non-linear prediction, phase synchronization, symbolization, recurrence quantification analysis (RQA), and functional connectivity analysis techniques (Marwan et al., 2013; Aguirre et al., 2018; Lombardi et al., 2019). Studies have demonstrated that information transfer between the cardiovascular and cardiorespiratory systems exhibits strong non-linearity (Novak et al., 1993), and therefore, linear approaches alone cannot fully quantify physiological and pathophysiological regulatory processes. There is no generally superior approach capable of considering all aspects of coupling analysis (linearity, non-linearity, causality, multivariate analysis, directionality, coupling strength) and its quantitative evaluation. Some of these approaches include one or more of these aspects, but usually not to a sufficient extent, so the time series with their mutual interactions and couplings can only be interpreted and analyzed incompletely and in parts. Furthermore, many of these approaches are not standardized and not user-friendly (degrees of freedom, preconditions, model selection and model order estimation, scale dependency). They are based on purely mathematical concepts, making it difficult to select the “right” approach to apply them to quantify physiological and pathophysiological regulatory processes.

Computerized quantitative models are essential to integrating and evaluating the information generated by these methods. For example, the Physiome Project provides a framework for qualitatively evaluating such information. The term “Physiome” is derived from “physio,” meaning “life,” and “ome,” meaning “as a whole,” (Leem, 2016). The project developed a multiscale modeling framework to understand physiological functions, enabling models to be combined and linked hierarchically.

In this study, we employed the High-Resolution Joint Symbolic Dynamics (HRJSD) approach, which represents an enhanced version of the classical Joint Symbolic Dynamics (JSD). The HRJSD approach was initially developed for the analysis of non-linear cardiovascular and cardiorespiratory couplings in acute schizophrenia (Schulz et al., 2013b; Schulz et al., 2013c; Schulz et al., 2017a; Schulz et al., 2017b). This was based on analyzing dynamic processes through symbols (Voss et al., 1996). The HRJSD is founded upon a symbolization procedure that enables a coarse-grain quantitative evaluation of the classification and characterization of short-term regulatory bivariate coupling patterns that are predominant in the interaction generated by the ANS. The HRJSD employs a redundancy reduction strategy that groups single-word types into eight pattern families, thereby enabling a comprehensive quantification of bivariate short-term autonomic coupling patterns. Based on the concept of frequent deterministic pattern classification, the bivariate redundancy reduction strategy overcomes the limitations of classical univariate symbolization strategies. It facilitates a transition between univariate and bivariate symbolic analyses, significantly advancing over the standard JSD and Symbolic Coupling Traces (SCT) (Wessel et al., 2009, 2011). The HRJSD method overcomes the issues encountered by JSD in differentiating between decreases and steady states, as well as between small and large changes in autonomic regulation due to threshold level l = 0 and the alphabet A = {0,1} for symbol transformation. It is impossible to differentiate between noise, artifacts (for example, those generated by undersampling or ectopic events), and fluctuations arising from (patho) physiological regulatory processes when using JSD. However, both approaches have the main advantages of being insensitive to non-stationary time series and capable of capturing non-linear couplings through a straightforward procedure.

Therefore, the main objective of this study was to develop new directionality indices derived from coupling approaches that are based on simple mathematical principles, such as symbolization, and simple calculation procedures, enabling a comprehensive understanding of the underlying couplings in a fast and easy way, and do not have the limitations of already established approaches. Here, we developed new directionality indices enabling the assessment of coupling directions in bivariate and multivariate systems based on the high-resolution joint symbolic dynamics approach (HRJSD).

## 2 Materials and methods

### 2.1 Basics of high-resolution joint symbolic dynamics – HRJSD

Baumert et al. (2002) developed the joint symbolic dynamics (JSD) method to analyze nonlinear couplings between systolic blood pressure (SP) and heart rate (BBI) time series, relying on the analysis of dynamic processes using symbols (Kurths et al., 1995).

Schulz et al. (2013c) introduced an enhanced version of the classical JSD to overcome their restrictions: high-resolution joint symbolic dynamics (HRJSD), which is based on three symbols and a symbol-transformation threshold, which can be used to quantify short-term non-linear coupling by means of symbols.

Therefore, the direct analysis of successive signal amplitudes is based on discrete states (symbols). In short, HRJSD works by transforming the two investigated time series (here: BBI and SP) into symbol sequences based on their signal amplitudes using a given alphabet *A* = {0, 1, 2}. The bivariate sample vector *X* (Equation 1) of the two-time series with *x*_BBI_ and *x*_SP_ is transformed into a bivariate symbol vector *S* (Equation 2), where *n* are the nth beat-to-beat values of BBI and SP, respectively.


(1) $$X={\left\{{\left[x_{n}^{BBI},x_{n}^{SP}\right]}^{T}\right\}}_{n=0,1,\ldots }x\in R\;\left(\begin{array}{l} R\;\mathrm{is the subset of}\;\\ \mathrm{real positive numbers} \end{array}\right)$$

(2) $$S={\left\{{\left[s_{n}^{BBI},s_{n}^{SP}\right]}^{T}\right\}}_{n=0,1,\ldots }s\in 0,1,2$$

The bivariate symbol vector *S* is defined using the following definitions (Equations 3, 4):


(3) $$S_{n}^{BBI}=\{\begin{array}{l} 0:\left(x_{n+1}^{BBI}-x_{n}^{BBI}\right)<-l^{BBI}\\ 1:-l^{BBI}\leq \left(x_{n+1}^{BBI}-x_{n}^{BBI}\right)\leq l^{BBI}\\ 2:\left(x_{n+1}^{BBI}-x_{n}^{BBI}\right)>l^{BBI} \end{array}$$

(4) $$S_{n}^{SP}=\{\begin{array}{l} 0:\left(x_{n+1}^{SP}-x_{n}^{SP}\right)<-l^{SP}\\ 1:-l^{SP}\leq \left(x_{n+1}^{SP}-x_{n}^{SP}\right)\leq l^{SP}\\ 2:\left(x_{n+1}^{SP}-x_{n}^{SP}\right)>l^{SP} \end{array}$$

Increasing values were coded as “2,” decreasing values as “0,” and unchanging (no or little variability) values as “1,” respectively. Afterward, *S* was subdivided into short words (sequences of symbols, bins) *w_k_* of length *k* = 3. In this study, an adapted threshold *for* the individual physiological dynamic variability was equal to 25% of the standard deviation of the time series. The derived different word types from the BBI (*w*_BBI_) and SP ( $w_{SP}$ ) time series (word types ranging from: 000, 001,…, 221, 222) were organized into a normalized 27 × 27 vector matrix *W_n_* ranging from word type (000,000)^T^ to (222,222)^T^. These single-word types $w_{BBI},w_{SP}$ (total number of all word type combinations 27 × 27 = 729) were afterward grouped into eight pattern families’ *wf,* whereby the sum of probabilities of all single-word family occurrences *p*(*wf*) was normalized to 1. The eight pattern families (E0, E1, E2, LU1, LD1, LA1, P, V) describe different aspects of autonomic modulation of the BBI- and SP time series and were sorted into an 8 × 8 pattern family density matrix *Wf,* resulting in 64 coupling patterns. The pattern famines are defined as follows:

- *E0*, *E1,* and *E2*: Words consisting of three equal symbols (no variation of symbols) of type ‘0,’ ‘1’, and ‘2,’ respectively.
- *LU1* and *LD1*: Words consisting of two different symbols with low increasing behavior (LU1) and low decreasing behavior (LD1).
- *LA1*: Words consisting of two different alternating symbols of type ‘0’ and ‘2’ with an increasing-decreasing behavior.
- *P* and *V*: Words consisting of three different symbols with peak-like behavior (P) and valley-like behavior (V) (Schulz et al., 2013c)

From the word distribution density matrix *Wf,* the normalized joint probability of the occurrence of each word was estimated (Schulz et al., 2013b; Schulz et al., 2015).

**Figure 1:**
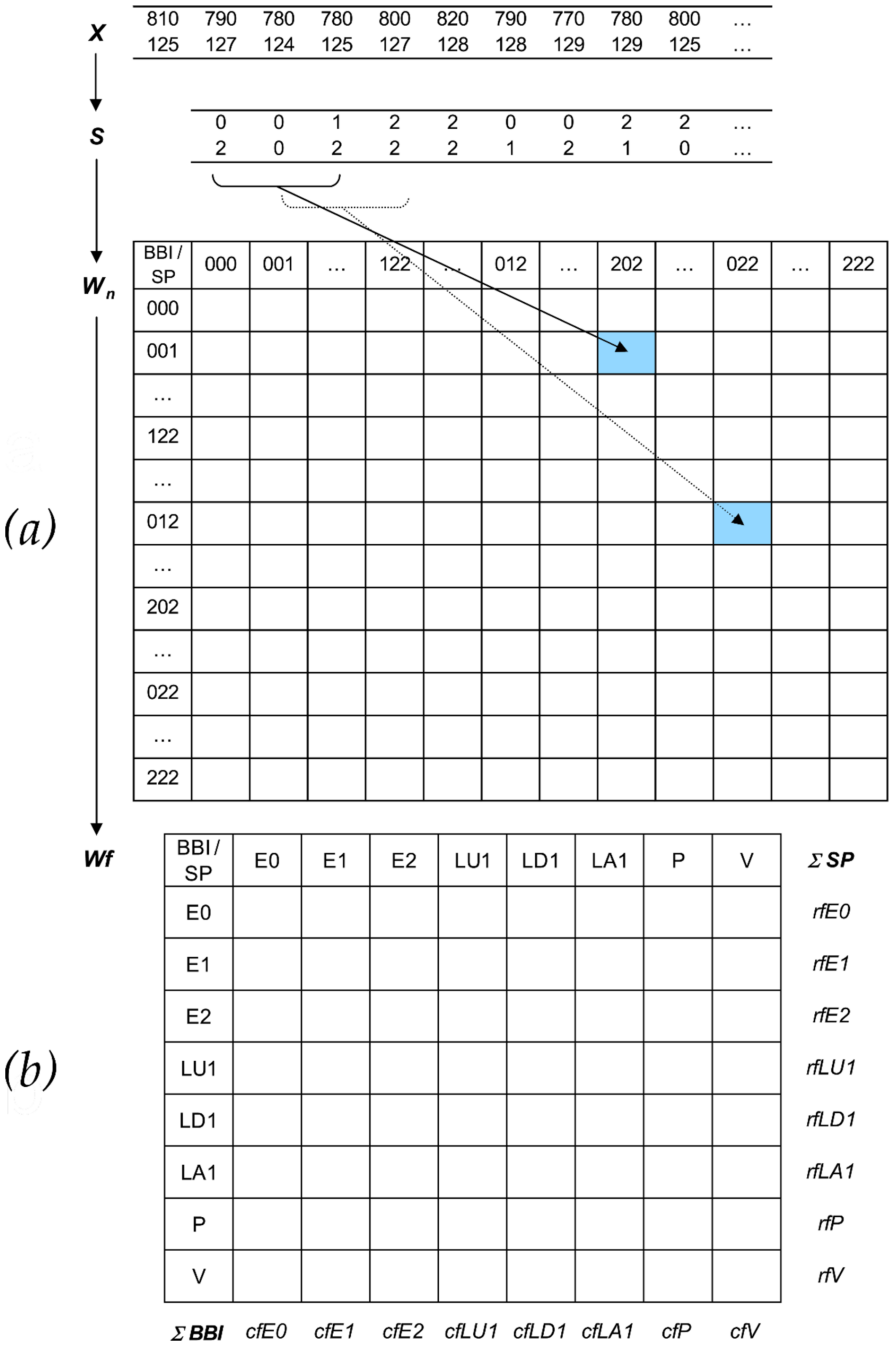
Basic principle of HRJSD. **(a)** Transformation of the bivariate sample vector *X* (BBI = beat-to-beat intervals [msec]; SP = systolic blood pressure [mmHg]) into the bivariate symbol vector *S* (0: decreasing values, 1: equal values, 2: increasing values) and word distribution density matrix *W*_n_ (27 × 27). **(b)** Word pattern family distribution density matrix *Wf* (8 × 8) with eight pattern families *wf* created from 27 single-word types *w*_BBI, SP_. Rows represent pattern families of BBI interval changes, and column pattern families of SP changes, *rf*_BBI_ (row): sum of the specific word family, *cf*_SP_ (column): sum of the specific word family.

From the matrix *Wf,* the sum of each (*n* = 8) column $cf_{SP}$ (*cf*E0, *cf*E1, *cf*E2, *cf*LU1, *cf*LD1, *cf*LA1, *cf*P, c*f*V) and the sum of each (*n* = 8) row *rf*_BBI_ (*rf*E0, *rf*E1, *rf*E2, *rf*LU1, *rf*LD1, *rf*LA1, *rf*P, *rf*V) were calculated (Figure 1).

### 2.2 Directionality index – bivariate system

**Figure 2:**
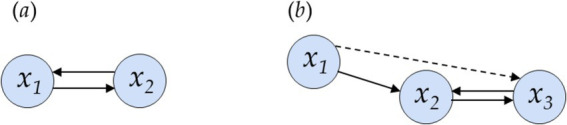
Examples of directional dependencies for direct and indirect couplings. Interdependence structure for **(a)** a bivariate and **(b)** a multivariate case. **(a)** Direct coupling exists for *x*_1_↔*x*_2_; **(b)** direct coupling exists for *x*_1_ → *x*_2_ and *x*_2_↔*x*_3_ and indirect coupling between *x*_1_ → *x*_3_ mediated by *x*_2_ (direction of coupling: →,← unidirectional, ↔ bidirectional). Adopted to Schulz et al. (2013a).

**Figure 3:**
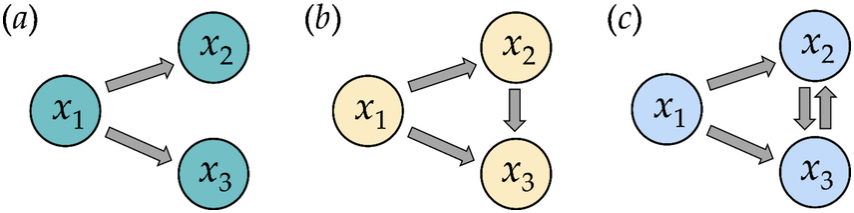
Simulated multivariate systems with their mutual influence between the time series *x*_1_, *x*_2_, and *x*_3_. Arrows indicating the causal coupling direction from one system to another (e.g. *x*_1_ → *x*_2_ means a unidirectional driving **(a, b)** from system 1 (*x*_1_) to system 2 (*x*_2_), and *x*_2_ → *x*_3_ means a bidirectional driving **(c)** between system 2 (*x*_2_) to system 3 (*x*_3_)).

To evaluate the physiological states of highly complex biological systems, it is important and necessary to determine synchronization processes within coupled complex systems and the predominant direction of their coupling. Causality can be defined by using the directionality of time to establish a causal ordering of dependent time series, encompassing both direct and indirect influences from one process to another. This definition can be applied to bivariate (two-time series, Figure 2A) and multivariate (more than two-time series, Figures 2B, 3) analysis. In the case of multivariate analysis, a distinction can be made between direct coupling (from one-time series to another) and indirect coupling (effects mediated through one or more other time series) (Schulz et al., 2013a).

The coupling direction can be determined, e.g., from the amplitudes of the system (properties of the system state) by calculating their mutual predictability (Schiff et al., 1996), from mutual nearest neighbors (Arnhold et al., 1999; Quiroga et al., 2000) in the reconstructed state space, or by applying information theoretical approaches (Schreiber, 2000; Palus et al., 2001; Palus and Stefanovska, 2003).

In the field of symbolization, so far, no approaches have been available to determine the coupling direction for bivariate or multivariate systems. The first attempts at this were integrated into Symbolic Coupling Traces (SCT) (Wessel et al., 2011). SCTs can detect delayed couplings (time lags) but cannot assess the coupling direction or the driver-response relationships. To address this gap, we introduced a directionality index (*D*_HRJSD_) derived from the 8 × 8 pattern family density matrix *Wf* from the HRJSD approach. This index is able to determine the dominant coupling direction and assess the driver-response relationships in bivariate (*n* = 2) (Figure 2A) and multivariate (*n* = 3) systems (Figure 2B).

For a bivariate system (*x*,*y*), the columns *cf*_x_ (*n* = 8) and the rows *rf*_y_ (*n* = 8) from the matrix *Wf* (Figure 1) were used to calculate *D*_HRJSD_(*x*,*y*) (Equation 5):


(5) $$D_{\mathrm{HRJSD}}\left(x,y\right)=-\left(\overset{n}{\underset{i=1}{\sum }}\frac{cf_{x}\left(i\right)-rf_{y}\left(i\right)}{cf_{x}\left(i\right)+rf_{y}\left(i\right)}\right)/n$$

If *D*_HRJSD_(*x*,*y*) is positive, driving (→) from system 1 (*x*) to system 2 (*y*) predominates (Equation 6) and becomes negative for the opposite case (Equation 7).


(6) $$D_{\mathrm{HRJSD}}\left(x,y\right)>0;x\to y$$

(7) $$D_{\mathrm{HRJSD}}\left(x,y\right)<0;y\to x$$

#### 2.2.1 Simulated coupled linear and non-linear systems to validate *D*_HRJSD_

Simulated data were used to validate *D*_HRJSD_. Therefore, two different multivariate models were applied (Baccala and Sameshima, 2001; Montalto et al., 2014), each with 100 simulated time series:

- Linear time series with a normal distribution of the variables, generated by a linear Gaussian AR model and
- Non-linear time series generated by a non-linear Gaussian AR model.

Three distinct multivariate coupled systems were generated for both linear and nonlinear models, incorporating varying mutual influences (unidirectional and bidirectional) between the time series (Figure 3).

The following equations were used for the three linear Gaussian autoregressive models (Baccala and Sameshima, 2001; Montalto et al., 2014) (Equations 8–10):

Linear system 1, LS1 (Figure 3A):


(8) $$\begin{array}{c} x_{1}\left(n\right)=0.95\sqrt{2}x_{1}\left(n-1\right)-0.9025x_{1}\left(n-2\right)+w_{1}\left(n\right)\\ x_{2}\left(n\right)=-0.5x_{1}\left(n-1\right)+w_{2}\left(n\right)\\ x_{3}\left(n\right)=0.4x_{1}\left(n-2\right)+w_{3}\left(n\right) \end{array}$$

Linear system 2, LS2 (Figure 3B):


(9) $$\begin{array}{c} x_{1}\left(n\right)=0.95\sqrt{2}x_{1}\left(n-1\right)-0.9025x_{1}\left(n-2\right)+w_{1}\left(n\right)\\ x_{2}\left(n\right)=0.5x_{1}\left(n-2\right)+w_{2}\left(n\right)\\ x_{3}\left(n\right)=-0.4x_{1}\left(n-3\right)-0.2x_{2}\left(n-2\right)+w_{3}\left(n\right) \end{array}$$

Linear system 3, LS3 (Figure 3C):


(10) $$\begin{array}{c} x_{1}\left(n\right)=0.95\sqrt{2}x_{1}\left(n-1\right)-0.9025x_{1}\left(n-2\right)+w_{1}\left(n\right)\\ x_{2}\left(n\right)=0.5x_{1}\left(n-2\right)+0.4x_{3}\left(n-1\right)+w_{2}\left(n\right)\\ x_{3}\left(n\right)=-0.4x_{1}\left(n-3\right)-0.2x_{2}\left(n-2\right)+w_{3}\left(n\right) \end{array}$$

Where *w*_1_(*n*), *w*_2_(*n*), and *w*_3_(*n*) were drawn from Gaussian noise with zero mean and unit variance. For linear system 3, a closed loop from *x*_3_(*n*) back to *x*_2_(*n*) via a direct connection was integrated, with *x*_3_ as the predominant driver.

For the non-linear models (Montalto et al., 2014) (Equations 11–13), *x*_2_(*n*) was modified by a quadratic term of $x_{1}^{2}$ . Thus, the three linear model equations changed to:

Non-linear system 1, NLS1 (Figure 3A):


(11) $$\begin{array}{c} x_{1}\left(n\right)=0.95\sqrt{2}x_{1}\left(n-1\right)-0.9025x_{1}\left(n-2\right)+w_{1}\left(n\right)\\ x_{2}\left(n\right)=-0.5x_{1}^{2}\left(n-1\right)+w_{2}\left(n\right)\\ x_{3}\left(n\right)=0.4x_{1}\left(n-2\right)+w_{3}\left(n\right) \end{array}$$

Non-linear system 2, NLS2 (Figure 3B):


(12) $$\begin{array}{c} x_{1}\left(n\right)=0.95\sqrt{2}x_{1}\left(n-1\right)-0.9025x_{1}\left(n-2\right)+w_{1}\left(n\right)\\ x_{2}\left(n\right)=0.5x_{1}^{2}\left(n-2\right)+w_{2}\left(n\right)\\ x_{3}\left(n\right)=-0.4x_{1}\left(n-3\right)-0.2x_{2}\left(n-2\right)+w_{3}\left(n\right) \end{array}$$

Non-linear system 3, NLS3 (Figure 3C):


(13) $$\begin{array}{c} x_{1}\left(n\right)=0.95\sqrt{2}x_{1}\left(n-1\right)-0.9025x_{1}\left(n-2\right)+w_{1}\left(n\right)\\ x_{2}\left(n\right)=0.5x_{1}^{2}\left(n-2\right)+0.5x_{3}\left(n-1\right)+w_{2}\left(n\right)\\ x_{3}\left(n\right)=-0.4x_{1}\left(n-3\right)-0.2x_{2}\left(n-2\right)+w_{3}\left(n\right) \end{array}$$

Where *w*_1_(*n*), *w*_2_(*n*), and *w*_3_(*n*) were drawn from Gaussian noise with zero mean and unit variance. For non-linear system 3, a closed loop from *x*_2_(*n*) back to *x*_3_(*n*) via a direct connection was integrated, with *x*_2_ as the predominant driver.

### 2.3 Directionality index – multivariate system

No methodical approach based on symbolization allows the detection of the coupling direction in multivariate coupled systems. Rather, no coupling approach is available that can determine the primary driver, the secondary driver, and the dominant responder in multivariate weakly coupled systems. To overcome the limitation of analyzing bivariate couplings, only the HRJSD approach was adopted in a further step for the quantification of multivariate couplings – the multivariate High-Resolution Joint Symbolic Dynamics (mHRJSD) (Schulz et al., 2018). In short, mHRJSD works in that way, that the set of three investigated time series (e.g., X, Y, and Z) was transformed into symbol sequences based on their signal amplitudes using a given alphabet *A* = {0, 1, and 2}. The trivariate sample vector *X* of these time series, *x*^X^, *x*^Y^ and *x*^Z^, were then transformed into a trivariate symbol vector *S*, where *n* were the *n*th beat-to-beat values of X, Y, and Z, respectively. Here, all single-word types *w*_X, Y, and Z_ were grouped into eight pattern families’ *w_f_* whereby the probabilities of all single-word family occurrences *p*(*w_f_*) were also normalized to 1. These eight pattern families were sorted into an 8 × 8 × 8 pattern family density matrix *Wf*. Furthermore, from the matrix *Wf*, the sum of each (*n* = 8) *x*-, *y*-, and *z*-plane (*pf*_X_, *pf*_Y_, *pf*_Z_) as *pf*E0, *pf*E1, *pf*E2, *pf*LU1, *pf*LD1, *pf*LA1, *pf*P, and *pf*V were calculated describing how one family pattern in one time series is coupled with all other eight pattern families of the other two time series.

For the mHRJSD approach, the proposed directionality index was further extended to determine the dominant coupling direction and assess the driver-response relationships in multivariate (*n* = 3) systems.

For a multivariate system (*x*,*y*,*z*), the single-word family occurrences *p*(*w_f_*) from the x-, y- and z-plane (*pf*_X_, *pf*_Y_, *pf*_Z_) from the 8 × 8 × 8 pattern family density matrix *Wf* were used to calculate *D*_mHRJSD_ (Equations 14–16). Thereby, for each coupling pathway, one directionality index was calculated (e.g., two interacting time series: *x* and *y* with *z* as the covariate |). Thus, for the coupling between the time series *x* and *y* with covariate *z*, the directionality index (Equation 14) is defined as:


(14) $$D_{\mathrm{mHRJSD}}\left(x,y|z\right)=-\left(\overset{n}{\underset{i=1}{\sum }}\frac{pf_{x|z}\left(i\right)-pf_{y|z}\left(i\right)}{pf_{x|z}\left(i\right)+pf_{y|z}\left(i\right)}\right)/n$$

For the coupling between the time series *x* and *z* with covariate *y*, the directionality index (Equation 15) is defined as:


(15) $$D_{\mathrm{mHRJSD}}\left(x,z|y\right)=-\left(\overset{n}{\underset{i=1}{\sum }}\frac{pf_{x|y}\left(i\right)-pf_{z|y}\left(i\right)}{pf_{x|y}\left(i\right)+pf_{z|y}\left(i\right)}\right)/n$$

For the coupling between the time series *y* and *z* with covariate *x*, the directionality index (Equation 16) is defined as:


(16) $$D_{\mathrm{mHRJSD}}\left(y,z|x\right)=-\left(\overset{n}{\underset{i=1}{\sum }}\frac{pf_{y|x}\left(i\right)-pf_{z|x}\left(i\right)}{pf_{y|x}\left(i\right)+pf_{z|x}\left(i\right)}\right)/n$$

If *D*_mHRJSD_(*x*,*y*|*z*) is positive, driving (→) from system 1 (*x*) to system 2 (*y*) predominates (Equation 17) and becomes negative for the opposite case (Equation 18).


(17) $$D_{\mathrm{mHRJSD}}\left(x,y|z\right)>0;x|z\to y|z$$

(18) $$D_{\mathrm{mHRJSD}}\left(x,y|z\right)<0;y|z\to x|z$$

If *D*_mHRJSD_(*x*,*z*|*y*) is positive, driving (→) from system 1 (*x*) to system 2 (*z*) predominates (Equation 19) and becomes negative for the opposite case (Equation 20).


(19) $$D_{\mathrm{mHRJSD}}\left(x,\;z|y\right)>0;x|y\to z|y$$

(20) $$D_{\mathrm{mHRJSD}}\left(x,\;z|y\right)<0;z|y\to x|y$$

If *D*_mHRJSD_(*y,z*|*x*) is positive, driving (→) from system 1 (*y*) to system 2 (*z*) predominates (Equation 21) and becomes negative for the opposite case (Equation 22).


(21) $$D_{\mathrm{mHRJSD}}\left(y,z|x\right)>0;y|x\to z|x$$

(22) $$D_{\mathrm{mHRJSD}}\left(y,z|x\right)<0;z|x\to y|x$$

Thus, three indices were derived, which are subsequently used to determine the strongest driver and the most dominant responder in the overall system. Therefore, all three indices were compared to whether they were greater (+) or less than 0 (−) (Table 1). This means that if the index is greater than zero for the multivariate interaction, “+” is set; if the index is less than zero, “−” is set. Afterward, the sum of the three comparisons of the three directionality indices was determined and counted. If a time series is present twice (++ or −− or +− or −+) as a driver, it dominates the overall system and is determined as the primary driver **D_mHRJSD_, and if a time series is present only once (+ or −), then it is determined as the secondary driver *D_mHRJSD_ of the overall system, and the non-occurring time series is the dominant responder *^−^D*_mHRJSD_.

**Table 1:** Determination of the primary driver (**D_mHRJSD_), secondary driver (*D_mHRJSD_), and the dominant responder (^−^*D*_mHRJSD_) in a multivariate system derived from the directionality indices *D*_mHRJSD_(*x*,*y*|*z*), *D*_mHRJSD_(*x*,*z*|*y*), and *D*_mHRJSD_(*y*,*z*|*x*).

*D*_mHRJSD_(*x,y|z*)	*D*_mHRJSD_(*x,z|y*)	*D*_mHRJSD_(*y,z|x*)	**D_mHRJSD_	*D_mHRJSD_	*^−^D*_mHRJSD_
+ = *x*	+ = *x*	+ = *y*	*x*	*y*	*z*
− = *y*	− = *z*	− = *z*	*z*	*y*	*x*
+ = *x*	+ = *x*	− = *z*	*x*	*z*	*y*
− = *y*	− = *z*	+ = *y*	*y*	*z*	*x*
+ = *x*	− = *z*	− = *z*	*z*	*x*	*y*
− = *y*	+ = *x*	+ = *y*	*y*	*x*	*z*
+ = *x*	− = *z*	+ = *y*	*x-y-z*	*x-y-z*	*x-y-z*
− = *y*	+ = *x*	− = *z*	*x-y-z*	*x-y-z*	*x-y-z*

For exapmple: *D*_mHRJSD_(*x*,*y*|*z*) > 0, *D*_mHRJSD_(*x*,*z*|*y*) > 0, and *D*_mHRJSD_(*y*,*z*|*x*) > 0, resulting in (+++ equal to *x*-*x*-*y*) or *D*_mHRJSD_(*x*,*y*|*z*) < 0, *D*_mHRJSD_(*x*,*z*|*y*) > 0, and *D*_mHRJSD_(*y*,*z*|*x*) < 0, resulting in (−+− equal to *y*-*x*-*z*). The indices **D_mHRJSD_, *D_mHRJSD,_ and *^−^D*_mHRJSD_ are determined by their absolute values in descending order of importance of their values. For the first example (+++), we have information flows from *x* → *y*, *x* → *z,* and *y* → *z* resulting in *x* as **D_mHRJSD_, *y* as *D_mHRJSD,_ and *z* as *^−^D*_mHRJSD_. For the second example (−+−), we have information flows from *y* → *x*, *x* → *z,* and *z* → *y,* resulting in a closed-loop where **D_mHRJSD_, *D_mHRJSD,_ and *^−^D*_mHRJSD_ cannot be clearly determined (these options are represented by the last two lines in the Table 1**)**.

The simulated linear and non-linear AR systems were validated with two additional approaches: the normalized short-time partial directed coherence (NSTPDC) (Adochiei et al., 2013) and the multivariate transfer entropy (MuTE) (Montalto et al., 2014). Both methods allow us to determine the coupling direction. NSTPDC mainly detects linear coupling, whereas MuTE mainly detects non-linear coupling. In short, NSTPDC is based on an *m*-dimensional AR model with the order *p* and allows determining linear Granger causality in the frequency domain. Mute is an information-theoretical approach that detects the information transfer between multivariate joint processes and discovers purely non-linear interactions with a range of interaction delays. We applied in-house software in the programming environment MatlabR2013b.

## 3 Results

### 3.1 Results of simulated systems to validate *D*_HRJSD_

All three methods, HRJSD, NSTPDC, and MuTE, calculated a directionality index *D* (*D*_HRJSD_, *D*_NSTPDC_, *D*_MuTE_), which was used for validation. These three indices have in common that if the index is positive, driving (→) from system 1 (*x*) to system 2 (*y*) predominates and becomes negative for the opposite case that system 2 (*y*) is driving system 1 (*x*) (Table 2). Table 2 presents the results of the simulated linear and non-linear AR models with the underlying simulated driver-response relationships in a multivariate context and the results of the applied directionality indices.

**Table 2:** Results of simulated linear and non-linear autoregressive (AR) systems to validate the directionality index *D*_HRJSD_ (blue: driver variable).

Simulated driver-response relationship	Coupling	AR model	Directionality index		
			*D*_HRJSD_	*D*_NSTPDC_	*D*_MuTE_
**1** → **2**	Linear	Linear	0.013	2.0	1.0
**1** → **3**	Linear	Linear	0.052	2.0	1.0
**1** → **2**	Linear	Linear	0.012	2.0	1.0
**1** → **3**	Linear	Linear	0.028	2.0	1.0
**2** → **3**	Linear	Linear	0.012	1.8	0.7
**1** → **2**	Linear	Linear	0.011	2.0	1.0
**1** → **3**	Linear	Linear	0.012	2.0	1.0
**2** ⇄ **3**	Linear	Linear	−0.011	−0.5	−0.6
**1** → **2**	Non-linear	Non-linear	−0.037	1.0	1.0
**1** → **3**	Linear	Non-linear	0.106	2.0	1.0
**1** → **2**	Non-linear	Non-linear	−0.036	1.4	1.0
**1** → **3**	Non-linear	Non-linear	−0.019	2.0	1.0
**1** → **3**	Non-linear	Non-linear	−0.010	2.0	1.0
**1** → **2**	Non-linear	Non-linear	−0.030	1.5	1.0
**1** → **3**	Non-linear	Non-linear	−0.015	2.0	1.0
**2** ⇄ **3**	Non-linear	Non-linear	−0.002	1.5	0.8

For example, here, the first two rows are explained where the information flows were simulated with a linear AR model, linearly coupled variables, and simulated driver-response relationships 1 → 2 and 1 → 3 (blue represents the simulated driver variable). That means time series 1 transfers information to time series 2 and 3. The coupling directions are from 1 to 2 and 1 to 3.

Linear system 1:

**1** → 2 and **1** → 3: *D*_HRJSD_, *D*_NSTPDC_, and *D*_MuTE_ are positive; correct classification of the predominating coupling directions (**1** is the driver).

Linear system 2:

**1** → 2, **1** → 3, **2** → 3: *D*_HRJSD_, *D*_NSTPDC_, and *D*_MuTE_ are positive; correct classification of the predominating coupling directions (**1** and **2** are drivers).

Linear system 3:

**1** → 2, **1** → 3: *D*_HRJSD_, *D*_NSTPDC_, and *D*_MuTE_ are positive; correct classification of the predominating coupling directions (**1** is the driver).

2⇄**3**: *D*_HRJSD_, *D*_NSTPDC_, and *D*_MuTE_ are negative; correct classification of the predominating coupling direction (**3** is the driver).

For the linear AR model with purely linear couplings among the three variables (1, 2, 3), all directionality indices (*D*_HRJSD_, *D*_NSTPDC_, *D*_MuTE_) were able to correctly detect the predominating coupling directions and the related driver variable.

Non-linear system 1 (Table 2):

**1** → 2: *D*_NSTPDC_ and *D*_MuTE_ are positive; correct classification of the predominating coupling direction (**1** is the driver).

*D*_HRJSD_ is negative; incorrect classification of the predominating coupling direction. *D*_HRJSD_ detects variable 2 as the driver.

**1** → 3: *D*_HRJSD_, *D*_NSTPDC_, and *D*_MuTE_ are positive; correct classification of the predominating coupling direction (**1** is the driver).

Non-linear system 2:

**1** → 2, **1** → 3, **2** → 3: *D*_NSTPDC_ and *D*_MuTE_ are positive; correct classification of the predominating coupling directions (**1** and **2** are drivers).

*D*_HRJSD_ is negative; incorrect classification of the predominating coupling directions. *D*_HRJSD_ detects variables 2 and 3 as the drivers.

Non-linear system 3:

**1** → 2, **1** → 3, **2** ⇄3: *D*_NSTPDC_ and *D*_MuTE_ are negative; correct classification of the predominating coupling directions (**1** and **2** are drivers).

*D*_HRJSD_ is negative; incorrect classification of the predominating coupling directions. *D*_HRJSD_ detects variables 2 and 3 as the drivers.

For the non-linear AR model with purely non-linear couplings among the three variables (1, 2, 3), only NSTPDC and MuTE were able to correctly detect the predominating coupling directions and the related driver variable. *D*_HRJSD_ could partly detect the dominating coupling direction in non-linear systems (non-linear system 1).

The proposed directionality index *D*_HRJSD_ derived from the HRJSD approach is able to correctly detect the dominating coupling direction in linear bivariate coupled systems but is only partly able to detect the dominating coupling direction in non-linear bivariate coupled systems. Due to this limitation, in detailed investigations to determine the coupling direction, other methods should be used in addition to *D*_HRJSD_ (e.g., MuTE), which can also correctly determine the dominant driver-response relationships in pure non-linear systems.

### 3.2 Results of simulated systems to validate *D*_mHRJSD_

Similarly to validating *D*_HRJSD_ for the bivariate system, simulated data was used to validate *D*_mHRJSD_. Therefore, a multivariate linear Gaussian AR model was applied to generate a set of multivariate linear time series (*n* = 100) with a normal distribution of the variables (Baccala and Sameshima, 2001; Montalto et al., 2014). For the linear model, two different multivariate coupled systems were generated (Equations 8, 9) with different mutual influences (unidirectional, bidirectional) between the time series (Figures 3B,C). Non-linear AR models were not applied since *D*_HRJSD_ seems to only partly detect the correct driver-responder relationship between non-linear coupled time series.

The results of the two multivariate coupled linear AR systems showed that the determination of the multivariate directionality index *D*_mHRJSD_ works properly, as well as the determination of the primary driver **D_mHRJSD_, the secondary driver *D_mHRJSD,_ and dominant responder *^−^D*_mHRJSD_ in the multivariate systems (Table 3).

**Table 3:** Determination of the primary driver (**D_mHRJSD_), secondary driver (*D_mHRJSD_), and the dominant responder (^−^*D*_mHRJSD_) derived from the directionality indices *D*_mHRJSD_*(x,y|z)*, *D*_mHRJSD_*(x,z|y)*, and *D*_mHRJSD_*(y,z|x)* for two simulated multivariate coupled systems (LS2, LS3).

	*D*_mHRJSD_(*x_1_,x_2_|x_3_*)	*D*_mHRJSD_(*x_1_,x_3_|x_2_*)	*D*_mHRJSD_(*x_2_,x_3_|x_1_*)	**D_mHRJSD_	*D_mHRJSD_	*^−^D*_mHRJSD_
LS2	*x_1_*	*x_1_*	*x_2_*	*x_1_*	*x_2_*	*x_3_*
LS3	*x_1_*	*x_1_*	*x_3_*	*x_1_*	*x_3_*	*x_2_*

Linear system 2, LS2:

*x*_1_ → *x*_2_: *D*_mHRJSD_(*x*_1_,*x*_2_|*x*_3_) is positive; correct classification of the dominating coupling direction (*x*_1_ is driver).

*x*_1_ → *x*_3_: *D*_mHRJSD_(*x*_1_,*x*_3_|*x*_2_) is positive; correct classification of the dominating coupling direction (*x*_1_ is driver).

*x*_2_ → *x*_3_: *D*_mHRJSD_(*x*_2_,*x*_3_|*x*_1_) is positive; correct classification of the dominating coupling direction (*x*_2_ is driver).

From this results that:

*D*_mHRJSD_(*x*_1_,*x*_2_|*x*_3_) = *x*_1_ and *D*_mHRJSD_(*x*_1_,*x*_3_|*x*_2_) = *x*_1_ ⇒ **D_mHRJSD_ **=** *x*_1_.

*D*_mHRJSD_(*x*_2_,*x*_3_|*x*_1_) = *x*_2_ ⇒ *D_mHRJSD_ **=** *x*_2_.

⇒ *^−^D*_mHRJSD_ **=** *x*_3_.

For the coupled multivariate linear AR model (LS2), the correct driver-responder relationships were classified with *x*_1_ as the primary driver, *x*_2_ as the secondary driver, and *x*_3_ as the responder of the system, as it was simulated.

Linear system 3, LS3:

*x*_1_ → *x*_2_: *D*_mHRJSD_(*x*_1_,*x*_2_|*x*_3_) is positive; correct classification of the dominating coupling direction (*x*_1_ is driver).

*x*_1_ → *x*_3_: *D*_mHRJSD_(*x*_1_,*x*_3_|*x*_2_) is positive; correct classification of the dominating coupling direction (*x*_1_ is driver).

*x*_2_⇄*x*_3_: *D*_mHRJSD_(*x*_2_,*x*_3_|*x*_1_) is negative; correct classification of the dominating coupling direction (*x*_3_ is driver).

From this results that:

*D*_mHRJSD_(*x*_1_,*x*_2_|*x*_3_) = *x*_1_ and *D*_mHRJSD_(*x*_1_,*x*_3_|*x*_2_) = *x*_1_ ⇒ **D_mHRJSD_ **=** *x*_1_.

*D*_mHRJSD_(*x*_2_,*x*_3_|*x*_1_) = *x*_3_ ⇒ *D_mHRJSD_ **=** *x*_3_.

⇒ *^−^D*_mHRJSD_ **=** *x*_2_.

For the coupled multivariate linear AR model (LS3), the correct driver-responder relationships were classified with *x*_1_ as the primary driver, *x*_3_ as the secondary driver, and *x*_2_ as the responder of the system, as it was simulated.

The mHRJSD approach contains multivariate directionality indices *D*_mHRJSD_ (*D*_mHRJSD_(*x*,*y*|*z*), *D*_mHRJSD_(*x*,*z*|*y*), and *D*_mHRJSD_(*y*,*z*|*x*)), allowing us to determine the primary driver **D_mHRJSD_, the secondary driver *D_mHRJSD,_ and the dominant responder *^−^D*_mHRJSD_ in multivariate systems. Therefore, it has to be assumed that the time series to be analyzed are at least weakly coupled with each other. Limiting factors are that the proposed directionality index *D*_mHRJSD_ derived from the mHRJSD approach can only correctly detect the driver-responder relationships in linear coupled systems and cannot detect the driver-responder relationships in non-linear coupled systems. The mHRJSD approach can evaluate direct causal information transfer in multivariate systems. Despite this limitation of *D*_mHRJSD_, the feature to assess the driver-response relationships in multivariate systems is not implemented in any of the existing symbolization approaches and thus clearly complements the existing coupling approaches.

## 4 Discussion

The HRJSD approach emphasizes a clear characterization of how the couplings are composed by regulatory aspects of the ANS; it is able to quantify the coupling direction (directionality index: *D*_HRJSD_) in linear and non-linear coupled systems, which was not possible with existing symbolization approaches, neither for bivariate nor for multivariate systems, and assesses the driver-response relationships in bivariate (*n* = 2) and multivariate (*n* = 3) systems.

The newly developed directionality indices derived from (m)HRJSD are based on simple mathematical symbolization principles and simple calculation procedures, enabling a comprehensive understanding of the underlying couplings in a fast and easy way, and do not have the limitations of existing approaches. The main advantages of the (m) HRJSD-derived directionality indices are that they are insensitive to non-stationary time series; they are able to capture couplings through a simple, fast, and easy-to-implement symbolization procedure; they are scale-invariant; they are independent of time series length, model order selection, and significance level determination procedure. Moreover, clear advantages of using *D*_HRJSD_ over, e.g., MuTE are that with *D*_HRJSD,_ a multivariate interaction can be classified in the overall system in such a way that it can be determined which variable in the system generates the primary and secondary information flow and which variable only acts as a responder in the system. This assessment is currently only possible for linear systems. Further research with other models and time delays will provide further insight into *D*_HRJSD_. Most of the already established coupling approaches, e.g., partial directed coherence (PDC) or directed transfer function, depend on the reliability of the fitted multivariate autoregressive model (MAR) (i.e., optimal model order, epoch length) and a significance level has to be used for both to avoid spurious interactions (Schulz et al., 2013a). In general, most of these coupling approaches have high degrees of freedom, are not standardized in their preconditions (e.g., preprocessing steps, parameter settings, time-series length, model order selection, significance level determination, scale-independent data, and stationarity), and have been validated using physiological and pathophysiological cases (Schulz et al., 2013a).

The HRJSD approach includes different threshold levels and a directionality index, *D*_HRJSD_. The validation studies showed that the directionality index *D*_HRJSD_ is able to correctly detect the dominating coupling direction in linear coupled systems but is partly able to detect the dominating coupling direction in non-linear coupled systems. It is an intriguing observation that, within non-linear systems, where linear couplings may also prevail, the method can precisely detect these linear couplings. This suggests that the method can be employed to identify the part of the underlying coupling in a system, irrespective of its linear or non-linear nature and regardless of the type (linear or non-linear) of coupling between the variables (Table 2, NLS1, 1→3).

The HRJSD approach and other symbolization approaches are only able to analyze bivariate couplings, whereas the HRJSD approach is also able to determine the driver-responder relationship; facing this, the mHRJSD approach is able to quantify multivariate couplings and to determine dominant driver-responder relationship in multivariate coupled systems. These are outstanding new features for coupling analyses based on symbolizations. The mHRJSD approach facilitates multivariate analysis by incorporating a third time series, enabling the coarse-grained evaluation of time series dynamics. As outstanding and unique features of the mHRJSD approach are the implemented multivariate directionality indices *D*_mHRJSD_ (*D*_mHRJSD_(*x*,*y*|*z*), *D*_mHRJSD_(*x*,*z*|*y*), and *D*_mHRJSD_(*y*,*z*|*x*)) allowing us to determine the primary driver **D_mHRJSD_, the secondary driver *D_mHRJSD,_ and the dominant responder *^−^D*_mHRJSD_ in multivariate systems (assumption: weakly coupled system). The simulation procedure revealed that the proposed directionality index *D*_mHRJSD_ derived from the mHRJSD approach is able to correctly detect the driver-responder relationships in linear coupled systems. Moreover, the mHRJSD approach is able to evaluate the direct causal information transfer in multivariate systems.

However, given the limitation of *D*_HRJSD_ (e.g., MuTE) in fully determining coupling direction in nonlinear coupled systems, additional methods should be employed. The method-specific characteristics of the different coupling approaches operate in different domains in the assessment of coupling (strength and direction) and causality, and there is currently no superior approach that can combine all the advantages in a single approach. While non-linear methods study complex signal interactions, linear methods favor the frequency domain representation of biological signals (characterizing the connectivity between specific oscillatory components) (Schulz et al., 2013a).

Due to this restriction so far, it would be of great interest to test other models and see how the newly proposed directional indices are able to evaluate driver-response relationships. Here, the physiome as a computerized quantitative model could be an option. The physiome is the definitive quantitative and integrated description of the functional behavior of the physiological state of an individual or species. The physiome describes the physiological dynamics of the normal, intact organism. It is built upon information and structure, namely the genome, proteome, and morpheme. The physiome must define relationships from genome to organism and from functional behavior to gene regulation. Quantitative models in physiome are unlike any other database-driven research area, including bioinformatics, network biology, or big data analysis. The physiomic model is a repository of previous data and a tool for testing and predicting results by varying factors. Thereby, models can be defined at various levels of abstraction: the conceptual level, the mathematical level, the formulation level, and the solution level (Leem, 2016). Physiome and network physiology are very similar as they pursue integrative concepts. The multidisciplinary field of network physiology studies how different physiological systems and subsystems interact to regulate their functions. It looks at how these systems interact across different spatial and temporal scales, from cellular to organism levels. It also looks at how they communicate and work together to generate different physiological states and behaviors in health and disease (Bartsch et al., 2015b; Ivanov et al., 2016a; Ivanov, 2021).

The cardiovascular, cardiorespiratory, and central networks are intricate physiological systems with both direct and indirect interactions. In the investigation of these networks, bivariate approaches are frequently employed. Nevertheless, it seems reasonable to posit that multivariate approaches will become more prevalent in place of bivariate ones, given that they enhance the characterization of causal or non-causal interrelationships between the networks. For instance, multivariate coupling analysis, for example, heart rate, systolic blood pressure, respiration, and central activity, may provide more advanced information about the complex autonomic network in physiological and pathophysiological conditions than uni- and bivariate approaches.

For instance, interactions within the cardiorespiratory network are primarily reflected in respiratory sinus arrhythmia (RSA), characterized by rhythmic fluctuation in cardiac cycle intervals in relation to respiration. Two principal mechanisms have been proposed to explain the RSA. The first is the central influence of respiration on vagal cardiac motor neurons, and the second is the impact of respiration on intrathoracic pressure. In the context of central networks, the concepts of functional connectivity and neurophysiological brain processes are significant. These include bottom-up and top-down processing, whereby information flows from higher brain regions to peripheral end organ systems or vice versa (Schulz et al., 2013a; Schulz et al., 2018).

In further steps, the new directionality indices will be applied and tested based on already analyzed and existing clinical data (cardiovascular system, cardiorespiratory system, central autonomic network) (Schulz et al., 2016; Schulz et al., 2018; Schulz et al., 2019) and compared with other methods in terms of their classification rate and accuracy. Moreover, further testing is necessary to ensure the robustness of the new directionality indexes and how time delays in the simulated models, as well as within the calculation procedure, influence the directionality results and the driver-response relationships.

## Data Availability

The original contributions presented in the study are included in the article/supplementary material, further inquiries can be directed to the corresponding author.
